# Effect of Post-Stroke Rehabilitation on Body Mass Composition in Relation to Socio-Demographic and Clinical Factors

**DOI:** 10.3390/ijerph17145134

**Published:** 2020-07-16

**Authors:** Grzegorz Przysada, Ewelina Czenczek-Lewandowska, Justyna Wyszyńska, Aneta Weres, Joanna Baran, Andrzej Kwolek, Justyna Leszczak

**Affiliations:** 1 Institute of Health Sciences, Medical College, University of Rzeszów, 35-959 Rzeszów, Poland; g.przysada@interia.pl (G.P.); e.czenczek@univ.rzeszow.pl (E.C.-L.); justyna.wyszynska@onet.pl (J.W.); anetaweres.ur@gmail.com (A.W.); joannabaran.ur@gmail.com (J.B.); kwoleka@o2.pl (A.K.); 2 Clinical Provincial Hospital No. 2, 35-301 Rzeszów, Poland

**Keywords:** stroke, stroke rehabilitation, body composition, muscle mass, fat mass

## Abstract

*Background and objectives*: Stroke is one of the leading causes of morbidity, mortality and long-term adult disability. The aim of this study was to assess the changes in body mass composition in patients after stroke in connection with selected socio-demographic and clinical factors (sex, age, type of stroke and time from the first symptoms) following the rehabilitation process. *Materials and Methods*: The study group consisted of 100 post-stroke subjects who participated in a comprehensive rehabilitation program for a duration of five weeks. The measurements of body composition by a Tanita MC 780 MA analyser were performed on the day of admission to hospital, on the day of discharge (after 5 weeks) and 12 weeks after discharge from hospital. *Results*: It was shown that before rehabilitation (Exam I) in the study group there were significant differences in body composition relative to sex, age and time from stroke. The rates of fat mass % and visceral fat level decreased after rehabilitation (Exam II) in both males and females. Exam II, at the end hospital rehabilitation, showed lower levels of fat mass %, visceral fat level, as well as fat-free mass % and higher values of total body water % and muscle mass %. In Exam III, i.e., 12 weeks after discharge, all of the parameters retained their values. *Conclusions:* The study shows an association between stroke risk factors (primarily age, sex and time from the onset of the first symptoms of stroke) and body mass composition resulting from rehabilitation. The type of stroke and the effects of rehabilitation on body mass components showed no differences. Comprehensive rehabilitation had a positive effect on the body mass components.

## 1. Introduction

Stroke affects approximately 24–54% of society worldwide, and is one of the leading causes of mortality and disability. It is estimated that the problem annually affects 15 million people worldwide, and 1 in 3 stroke patients die as a result [1,2,3,4]. There are well-known factors contributing to the onset of stroke. It is known that above 55 years of age the risk of stroke doubles in each decade of life. There are also sex-related differences, as men are more likely to be affected; however, after 70 years of age this difference is no longer observed [5]. In the related literature the following non-modifiable factors have also been pointed out: environment, socioeconomic status, genetic determinants as well as ethnicity [6,7]. However, in stroke prevention, modifiable factors become the most important. The conditions most seriously contributing to the problem include abdominal obesity, which is preceded only by hypertension and smoking. The list also comprises unhealthy diet and sedentary lifestyle, associated with almost total physical inactivity. All of the above factors are present for over 80% of all the diagnosed cases of stroke [8,9,10,11].

Over the past few years it has been recognized that abdominal obesity is one of the factors adversely affecting the circulatory system. It has been established that body fat, most of all visceral fat (VFAT), is an important tissue with an endocrine function, responsible for the production of various active substances, including, most importantly, inflammatory mediators promoting the development of atherosclerosis. The unprecedented dynamic increase in the number of people with this problem worldwide results from the fact that the functioning of the cardiovascular system is overburdened in more than 50% of members of developed societies [12]. Data reported by the INTERHEART study show that abdominal obesity is the second and the third most important risk factor for heart attack and stroke, respectively. Numerous studies have also confirmed that there is a linear relationship between obesity and the risk of cardiovascular diseases in general [13]. A Physicians’ Health Study (PHS) pointed out that the risk of stroke is two times lower in the population with BMI < 23 kg/m^2^, compared to people with BMI > 30 kg/m^2^; [14,15]. However, BMI does not account for body composition; therefore, according to some authors, the measure is insufficient for assessing risk factors for stroke [16,17,18]. Given the above, it is necessary to assess body mass components and examine their relationship not only to the incidence of stroke, but also in the context of recovery. It has been established that comprehensive rehabilitation initiated as early as possible following a stroke incident enables, inter alia, faster increase in muscle mass, and consequently recovery of the lost functions and mobility [19]. After stroke, a decrease in muscle strength and a decrease in (PMM) muscle tissue content is observed in paresis limbs [20,21]. It is the opposite with fat content (FAT) after stroke, whose range increases [22,23]. Stroke causes a loss of fat-free body mass (FFM) and bone mineral content to a large extent on the paresis side. Studies by other authors indicate that, as time goes by, the percentage of body fat increases, but on the other hand lean tissue and muscle decrease [24]. This is associated with decreased physical activity. Therefore, there is a need for continuous and comprehensive rehabilitation in these patients.

The study was designed to assess the change of body mass composition components (body fat mass (FAT), fat-free body mass (FFM), visceral fat level (VFAT), muscle mass (PMM), body water (TBW)) in patients after stroke in connection with selected socio-demographic and clinical factors (i.e., sex, age, type of stroke, and time from the first symptoms of stroke) following rehabilitation.

## 2. Materials and Methods

This study was conducted in accordance with the ethical rules of the Helsinki Declaration, approved by the Local Bioethics Commission (Consent No. 2015/10/03). Written consent was obtained from all of the participants in the study.

### 2.1. Participants

The study was carried out in a group of 100 post-stroke subjects. They were recruited from patients in the Clinical Rehabilitation Ward of an Early Neurological Rehabilitation Unit in a hospital. The stroke was confirmed by CT and MRI examinations. The study group comprised 42 females and 58 males. They were divided into three age groups: 19–50 years (23 individuals), 51–65 years (42 individuals), 66–88 years (35 individuals). Ischemic stroke had been diagnosed in 82 and haemorrhagic stroke in 18 patients. Furthermore, the patients were divided relative to time from stroke onset: <6 months (28 subjects), 6–12 months (18 subjects), >12 months (54 subjects).

All of the post-stroke patients participated in a comprehensive rehabilitation program five days a week (from Monday to Friday) for five weeks at the rehabilitation clinic. The rehabilitation program was based on neuro-developmental treatment methods, gait and upper limb training, as well as exercise with equipment using biological feedback or static and dynamic parapodium. The first examination (Exam I) was conducted on admission to the clinic, before the rehabilitation started. The second examination (Exam II) was performed on discharge, after the five-week hospital-based rehabilitation. The third examination (Exam III) was carried out three months after discharge from the clinic, during a follow-up visit.

A total of 128 people participated in Exam I; however, after applying all of the inclusion and exclusion criteria to the study group, complete data from the three required examinations was obtained from a group of 100 patients.

The inclusion criteria were: diagnosis of stroke, complete first stroke, participation in the early hospital rehabilitation for minimum 4 weeks, ability to stand without assistance, ability to walk without aid, no impairments in higher mental functions and patient’s informed consent to participate in the study. Exclusion criteria were: lack of patient’s consent to participate in the study, incomplete stroke (e.g., Transient Ischemic Attack, TIA), second or subsequent stroke, lack of ability to stand without assistance (balance disorders and dizziness), ischemic lesion located in the cerebellum and brain stem, electronic implants, epilepsy, pregnancy, menstruation in females or leg injuries incurred following stroke onset.

### 2.2. Measurements

Body mass components were assessed with the Tanita MC 780 MA analyser, which operates based on electrical bio-impedance (BIA) measurements. The analyser is certified and approved for clinical use. It also has a certificate of compliance with 93/42 EEC (EU standard for medical equipment). Body composition was assessed for the contents of body fat mass (FAT%), fat-free mass (FFM level), visceral fat (VFAT%), muscle mass (PMM%) and body water (TBW%). Body height was measured with an accuracy up to 0.1 cm using a PORTSTAND 210 portable stadiometer. The measurements were performed in standard conditions. The subjects, in underwear and with no shoes, were instructed to assume a straight body posture.

### 2.3. Statistical Analysis

The analyses applied descriptive statistics (mean with 95% confidence interval, standard deviation, median, quartiles, minimum and maximum values). Analyses of differences between quantitative variables and independent nominal dichotomous variables were carried out using a t-test for independent samples or a Mann–Whitney test. A Kruskal–Wallis test was applied to assess the significance of the differences between the quantitative variables and variables of more than two categories. The choice of the tests depended on the normality of distributions of quantitative variables (verified with Kolmogorov–Smirnov and Shapiro–Wilk tests) and on the equal size of the groups of independent variables (verified with a chi-squared test). If the above assumptions were not met, non-parametric statistical methods were required. The significance level was assumed at α < 0.05. All of the calculations and statistical analyses were computed using STATISTICA ver. 10.0 (StatSoft, Kracow, Poland).

## 3. Results

**Figure 1:**
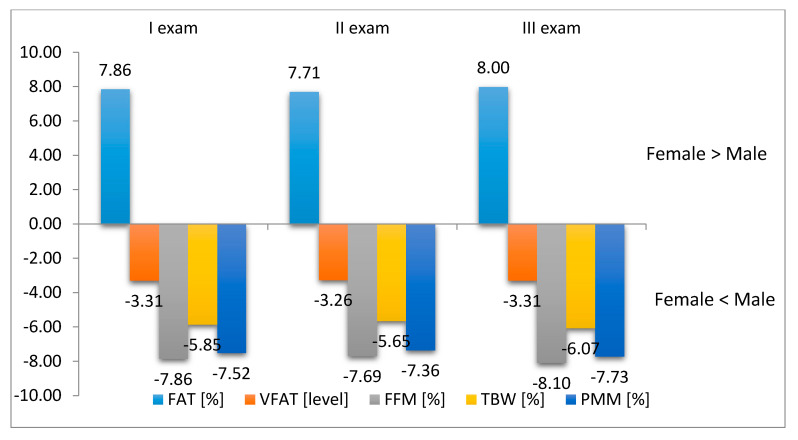
Difference between body mass components versus subjects’ sex.

It was shown that before rehabilitation (Exam I) in the study group there were significant differences in body composition relative to sex. With respect to FAT% rate and VFAT level it was possible to notice a decrease in these parameters following rehabilitation (Exam II) in both males and females. On the other hand there was an increase in TBW% and PMM% rates. It was observed that the values identified for the above parameters 12 weeks after the rehabilitation (Exam III) were at a similar level (Table 1, Figure 1).

**Figure 2:**
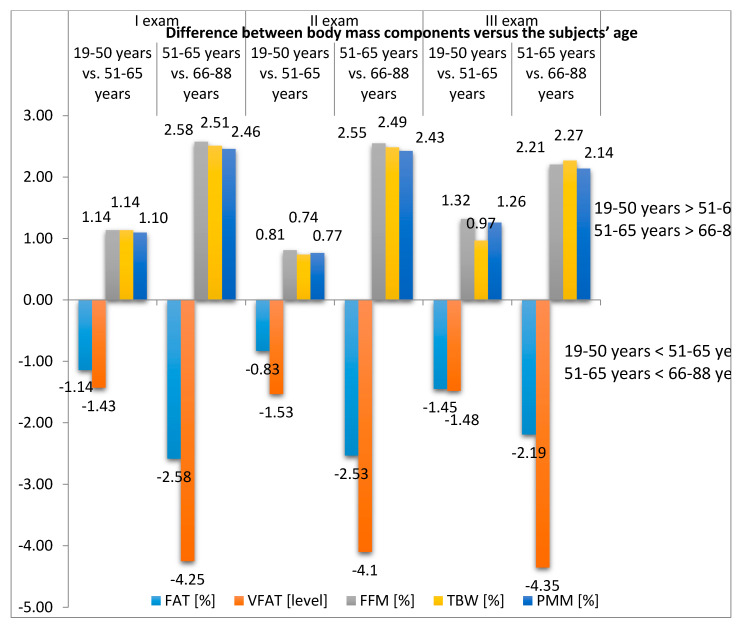
Difference between body mass components versus subjects’ age.

All of the age groups had decreased VFAT level at the end of the hospital-based rehabilitation (Exam II), and the rates were retained until the follow-up visit (Exam III) (Table 2, Figure 2).

**Figure 3:**
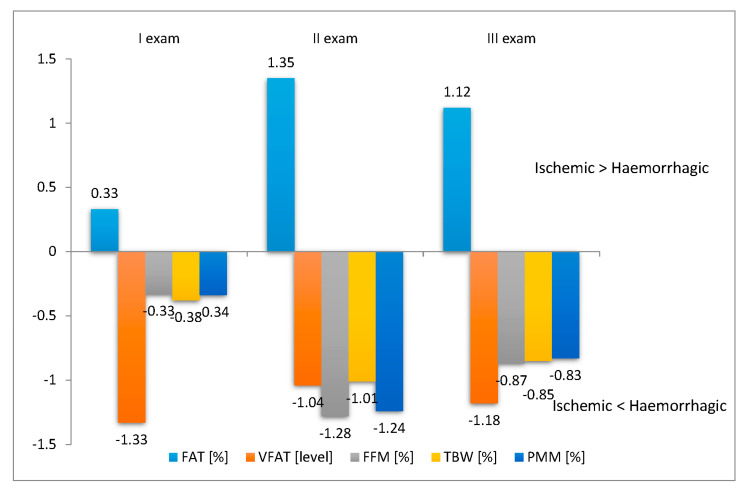
Difference between body mass components versus type of stroke.

Analyses taking into account the type of stroke and the effects of rehabilitation on body mass components showed no statistically significant differences relative to ischemic and haemorrhagic stroke. However, the contents of FAT% and VFAT level decreased at the end of hospital-based rehabilitation (Exam II) and the effect was retained at the follow-up 12 weeks after discharge from hospital (Exam III). There was an increase in PMM% content (Table 3, Figure 3).

**Figure 4:**
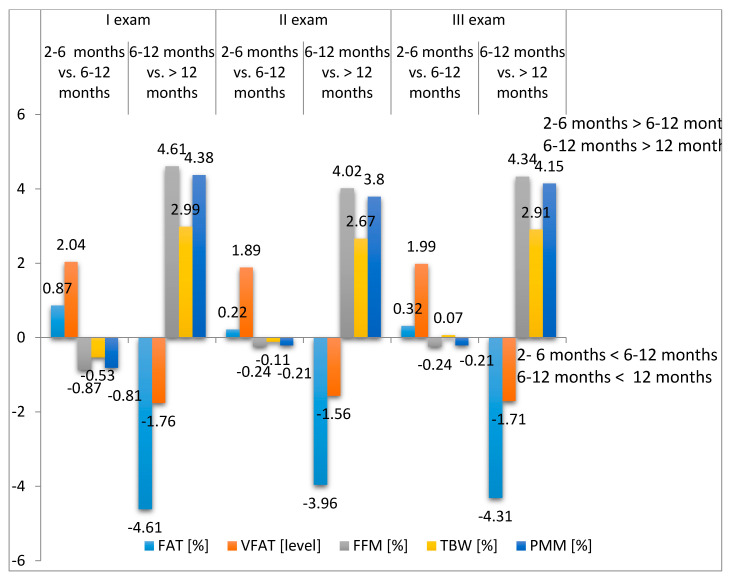
Difference between body mass components versus time from onset of the first stroke symptoms.

Taking into account the time from the onset of the first symptoms of stroke, statistically significant relationships were found for FAT%, FFM%, TBW% and PPM%, with respect to rehabilitation time. Exam II, at the end of the hospital rehabilitation, showed lower levels of FAT%, VFAT level and FFM% as well as higher values of TBW% and PMM%. In Exam III, all of the parameters retained their values (Table 4, Figure 4).

**Figure 5:**
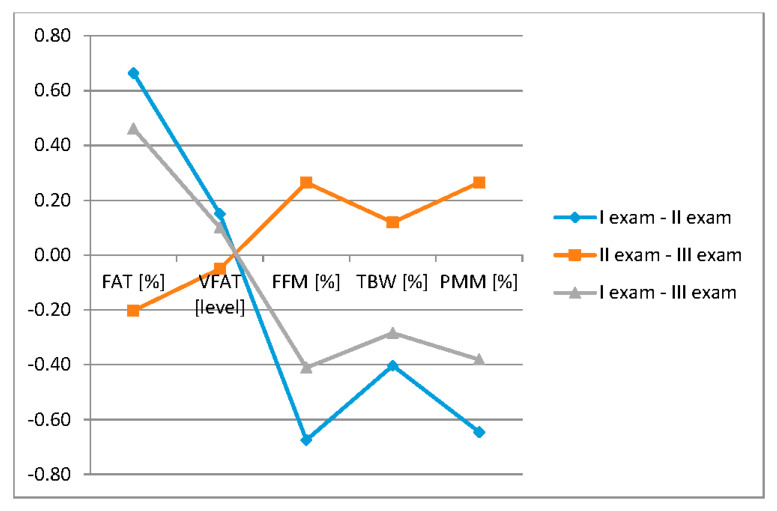
The changes of body mass components during the time.

Statistical differences were observed between Exam I and II for all body mass components (FAT, VFAT, FFM, TBW, PMM), and in Exam I and III for FAT, FFM, TBW, PMM (Table 5, Figure 5).

Multifactorial analysis showed that significant results were observed with regard to sex in Exams I and III among all body weight components, for age in FAT and VFAT during all studies, for time from stroke in FAT in Exam III, VFAT during all exams and FFM and PMM during Exam III. No statistically significant results were found for: sex and type of stroke; age and type of stroke; age and time from stroke; type of stroke and time since stroke; gender, age and type of stroke; gender, age and time since stroke (Table 6).

## 4. Discussion

According to numerous sources, comprehensive rehabilitation significantly contributes to a decrease in mortality due to cardiovascular causes by as much as 20–25%. Rehabilitation improves physical efficiency, including in subjects with excessive weight and obesity, it reduces the progress of atherosclerosis and ultimately leads to faster recovery and improved quality of life [25,26]. However, patients with higher BMI are less likely to demonstrate an improvement in functional efficiency [27]. It is estimated that as many as 36% of first-ever stroke patients can be classified as clinically obese [28].

It has been proven that exercise and physical activity lead to increased metabolism of adipose tissue [29]. The current study confirmed that following rehabilitation there was a positive change in the content of FAT% and at the same time a significant increase in FFM% in all of the patients, which indicate a better return to functional efficiency. Numerous studies support the claim that total body fat and distribution of adipose tissue play important roles in the incidence of stroke as well as in the recovery process after stroke [30]. This was shown, for example, by Orsatti et al. in a study conducted in a group of menopausal women [31]. Importantly, increase in abdominal obesity led mainly to higher visceral fat, and higher incidence of stroke and cardiovascular disorders in the study group [32,33]. It has been established that unfavourable body fat distribution increases the likelihood of extreme obesity and cardiovascular problems. This is particularly visible at BMI 33.0 ± 0.6 kg/m^2^, which was confirmed, for example, by a study carried out by You et al. [34]. On the other hand, a study carried out by Park and Lee showed that comprehensive rehabilitation based on a 12-week weight reduction program leads directly to a greater loss of visceral fat, accompanied with a decrease in blood pressure and a decrease in glucose concentration, compared to a total reduction of subcutaneous fat and body weight by means of regular analysis of BMI, where statistically significant differences were not observed [35,36]. This problem was also investigated by other researchers who established that a high level of visceral body fat is of the greatest importance in the incidence of circulatory system disorders. The authors also assessed the visceral fat content in their research because its elevated level negatively affects the functioning of organs, and may contribute to the development of atherosclerosis, which in turn may be one of the causes of ischemic stroke due to insufficient blood flow through the vessels. Taking into account the patients’ age and sex it was found that this factor was not related to the psycho-demographic characteristics of the subjects. Presence of central obesity leads to a higher content of visceral fat; on the other hand, comprehensive weight reduction programs beneficially affect its level in the body, which is also suggested by the study reported by Staiger et al. [37]. Similar relationships were observed in our own research. The level of visceral fat after hospital rehabilitation (Exam II) decreased and its value remained stable up to 3 months after leaving the hospital.

Rehabilitation led to a significant increase in the patients’ muscle mass, which leads to greater efficiency. Skeletal muscles are considered as the most important effectors of disability after stroke. Most stroke survivors experience secondary changes in the skeletal muscles, i.e., decreases in muscle mass and increases in intramuscular fat, which are not beneficial for good functional outcome and gait independence [38,39]. Taking into account this aspect, regular and early hospital rehabilitation, and later rehabilitation at home, explains the higher rate of muscle mass increase identified in Exam II and the fact that it was maintained 12 weeks following discharge from hospital. Increase in muscle mass in patients with stroke, also observed by other researchers, ultimately contributes to improved articular mobility and quality of life in subjects after stroke. Therefore, it should be emphasized that well-planned and interdisciplinary post-stroke rehabilitation should be seen as a one of the main components of treatment and it leads to significant improvement of physical fitness recovery [40,41].

Another important issue in the prevention and treatment of strokes is body hydration. Chan et al. showed a significant relationship between water intake and the risk of heart disease. The 6-year research program took into account 8280 males and 12,017 females and showed that the risk of heart disease decreased by 41% in women and by 54% in men who drank at least 1.25l of water per day, compared to those with low water intake (half of the recommended daily water intake). It was concluded that regular hydration of the body correlates with decreased risk of cardiovascular diseases [42]. The level of hydration of the body is equally important in the process of improving and regaining health after stroke. Research suggests that stroke survivors who are dehydrated may have worse short-term functional outcomes [43].

Consumption of water is generally regarded as beneficial for health and is recommended to patients with stroke and obesity or excessive weight. Numerous studies show that increase in water intake is an effective way to decrease the risk of chronic diseases, and directly leads to body weight reduction, with simultaneous decrease of body fat [44]. Regular consumption of water leads to lower energy intake, which contributes to improved fitness of the subjects. It was also observed that if water is introduced into the diet, total daily energy intake is decreased by 10–13% [45]. Compared to other fluids, water lacks microelements and does not contribute to insulin release. Anton et al. demonstrated a faster rate of fat oxidation in people who are physically active and drink water, compared to those consuming energy drinks, juices or other fluids during low or moderate intensity exercise [46]. One of the studies reports that fat oxidation in such a situation was nearly 40% greater compared to other fluids. Similarly, data reported by Vij et al. suggest that water consumption leads to an increased rate of metabolic processes, contributing to body weight loss, or, as shown by Dennis et al., to its rapid reduction [47,48]. It can be concluded that sufficiently high water intake is conducive to better parameters of body mass components and at the same time to better effects of rehabilitation.

BMI alone is not sufficient to assess obesity because BMI does not take into account fat distribution as a measure of overall obesity. Thus, people with excess body fat cannot be distinguished from people with high muscle mass. Therefore, a risk estimation associated with obesity will be made in people with high muscle mass if only BMI is considered [16]. It is necessary to conduct further research focusing on body mass components including elevated body fat, visceral fat, muscle mass and body water in connection with the effects of rehabilitation [49].

## 5. Conclusions

Comprehensive rehabilitation had a positive effect on the decrease of FAT% rate, VFAT level and FFM% and increase in TBW% and PMM% rates. The level of body mass components achieved as a result of rehabilitation is sustained for 12 weeks after discharge from hospital.

## 6. Limitations of this Study

Selected socio-clinical parameters were taken into account in the study. There are more factors, including environmental factors, that may affect the body mass of the subjects and therapeutic effects. Among others, these are smoking, nutritional status, chronic comorbidities or pharmacotherapy. Undoubtedly, these factors should be included in the continuation of research on this topic.

## Figures and Tables

**Table 1:** Body mass components versus subjects’ sex.

Exam	Parameters	Sex				*p*
		Female		Male		
		*N* 42		*N* 58		
		Mean (95% Cl)	SD	Mean (95% Cl)	SD	
I	FAT [%]	30.98 (28.75–33.21)	7.15	23.12 (21.67–24.57)	5.51	**<0.001**
	VFAT [level]	8.10 (7.01–9.18)	3.48	11.41 (10.15–12.68)	4.82	**<0.001**
	FFM [%]	69.02 (66.79–71.25)	7.15	76.88 (75.43–78.33)	5.51	**<0.001**
	TBW [%]	48.79 (47.20–50.38)	5.10	54.64 (53.48–55.80)	4.42	**<0.001**
	PMM [%]	65.52 (63.40–67.64)	6.80	73.04 (71.67–74.41)	5.22	**<0.001**
II	FAT [%]	30.23 (28.05–32.41)	7.00	22.52 (21.06–23.98)	5.54	**<0.001**
	VFAT [level]	7.98 (6.90–9.05)	3.45	11.24 (9.99–12.49)	4.76	**<0.001**
	FFM [%]	69.79 (67.61–71.98)	7.02	77.48 (76.02–78.94)	5.55	**<0.001**
	TBW [%]	49.31 (47.75–50.87)	5.01	54.96 (53.79–56.13)	4.46	**<0.001**
	PMM [%]	66.26 (64.18–68.34)	6.67	73.62 (72.24–75.00)	5.26	**<0.001**
III	FAT [%]	30.60 (28.50–32.71)	6.77	22.60 (21.05–24.15)	5.89	**<0.001**
	VFAT [level]	8.00 (6.97–9.03)	3.32	11.31 (10.01–12.61)	4.94	**<0.001**
	FFM [%]	69.29 (67.24–71.33)	6.56	77.39 (75.85–78.94)	5.88	**<0.001**
	TBW [%]	48.95 (47.49–50.40)	4.67	55.02 (53.81–56.23)	4.60	**<0.001**
	PMM [%]	65.78 (63.84–67.72)	6.23	73.51 (72.04–74.97)	5.58	**<0.001**

*N*: number of subjects; M: mean; SD: standard deviation; FAT: body fat; VFAT: visceral fat; FFM: fat-free mass; TBW: total body water; PMM muscle mass; *p*: Mann–Whitney test; normal distribution (Kołmogorow–Smirnow test, Shapiro–Wilk test); bold value: statistical significance.

**Table 2:** Body mass components versus subjects’ age.

Exam	Parameters	Age						*p*
		19–50 Years		51–65 Years		66–88 Years		
		*N* 23		*N* 42		*N* 35		
		Mean (95% Cl)	SD	Mean (95% Cl)	SD	Mean (95% Cl)	SD	
I	FAT [%]	24.64 (21.96–27.32)	6.20	25.78 (23.65–27.90)	6.83	28.36 (25.50–31.23)	8.33	0.209
	VFAT [level]	7.43 (5.38–9–49)	4.75	8.86 (7.62–10.09)	3.95	13.11 (11.93–14.30)	3.45	**<0.001**
	FFM [%]	75.36 (72.68–78.04)	6.20	74.22 (72.10–76.35)	6.83	71.64 (68.77–74.50)	8.33	0.209
	TBW [%]	53.94 (51.80–56.07)	4.94	52.80 (51.26–54.34)	4.95	50.29 (48.20–53–39)	6.09	0.067
	PMM [%]	71.59 (69.04–74.13)	5.89	70.49 (68.47–72.52)	6.50	68.03 (65.30–70.75)	7.92	0.213
II	FAT [%]	24.23 (21.61–26–85)	6.06	25.06 (23.01–27–12)	6.59	27.59 (24.69–30.49)	8.45	0.233
	VFAT [level]	7.26 (5.19–9.34)	4.80	8.79 (7.57–10.00)	3.89	12.89 (11.72–14.05)	3.38	**<0.001**
	FFM [%]	75.77 (73.15–78.39)	6.06	74.96 (72.90–77–02)	6.61	72.41 (69.51–75.31)	8.45	0.239
	TBW [%]	54.03 (51.88–56–17)	4.96	53.29 (51.82–54–77)	4.73	50.80 (48.67–52.89)	6.19	0.065
	PMM [%]	71.97 (69.49–74.46)	5.75	71.20 (69.24–73.15)	6.27	68.77 (66.01–71.53)	8.05	0.238
III	FAT [%]	24.08 (21.20–26.95)	6.65	25.53 (23.42–27.63)	6.75	27.72 (24.84–30.59)	8.36	0.232
	VFAT [level]	7.26 (5.17–9.36)	4.85	8.74 (7.52–9.96)	3.91	13.09 (11.92–14.25)	3.39	**<0.001**
	FFM [%]	75.78 (72.98–78.57)	6.47	74.46 (72.37–76.55)	6.70	72.25 (69.37–75.13)	8.38	0.243
	TBW [%]	54.01 (51.73–56.29)	5.27	53.04 (51.56–54.52)	4.74	50.77 (48.65–52.89)	6.18	0.112
	PMM [%]	71.98 (69.33–74.64)	6.14	70.72 (68.73–72.71)	6.38	68.58(65.85–71–31)	7.94	0.233

*N*: number of subjects; M: mean; SD: standard deviation; FAT: body fat; VFAT: visceral fat; FFM: fat-free mass; TBW: total body water; PMM muscle mass; *p*: Kruskal–Wallis test; (Kołmogorow–Smirnow test, Shapiro–Wilk test); bold value: statistical significance.

**Table 3:** Body mass components versus type of stroke.

Exam	Parameters	Type of Stroke				*p*
		Ischemic		Haemorrhagic		
		*N* 82		*N* 18		
		Mean (95% Cl)	SD	Mean (95% Cl)	SD	
I	FAT [%]	26.48 (24.83–28.12)	7.44	26.15 (22.63–29.67)	7.07	0.886
	VFAT [level]	9.78 (8.78–10.78)	4.54	11.11 (8.73–13.49)	4.79	0.224
	FFM [%]	73.52 (71.88–75.15)	7.44	73.85 (70.33–77.37)	7.07	0.886
	TBW [%]	52.12 (50.89–53.34)	5.59	52.50 (49.85–55.15)	5.33	0.660
	PMM [%]	69.82 (68.26–71.37)	7.08	70.16 (66.82–73.51)	6.73	0.879
II	FAT [%]	26.00 (24.39–27.61)	7.33	24.65 (21.17–28.12)	6.98	0.445
	VFAT [level]	9.68 (8.69–10.68)	4.52	10.72 (8.40–13.05)	4.68	0.313
	FFM [%]	74.02 (72.41–75.63)	7.33	75.30 (71.79–78.81)	7.06	0.476
	TBW [%]	52.41 (51.20–53.61)	5.50	53.42 (50.78–56.06)	5.30	0.430
	PMM [%]	70.30 (68.77–71.84)	6.97	71.54 (68.20–74.87)	6.71	0.476
III	FAT [%]	26.16 (24.52–27.81)	7.48	25.04 (21.48–28.59)	7.15	0.548
	VFAT [level]	9.71 (8.71–10.70)	4.52	10.89 (8.39–13.39)	5.03	0.316
	FFM [%]	73.83 (72.20–75.47)	7.46	74.70 (71.24–78.15)	6.95	0.584
	TBW [%]	52.32 (51.09–53.54)	5.59	53.17 (50.58–55.75)	5.19	0.409
	PMM [%]	70.11 (68.56–71.67)	7.08	70.94 (67.65–74.23)	6.61	0.578

*N*: number of subjects; M: mean; SD: standard deviation; FAT: body fat; VFAT: visceral fat; FFM: fat-free mass; TBW: total body water; PMM muscle mass; *p*: Mann–Whitney test; (Kołmogorow–Smirnow test, Shapiro–Wilk test).

**Table 4:** Body mass components versus time from onset of the first stroke symptoms.

Exam	Parameters	Time from Stroke						*p*
		2–6 Months		6–12 Months		>12 Months		
		*N* 28		*N* 18		*N* 54		
		Mean (95% Cl)	SD	Mean (95% Cl)	SD	Mean (95% Cl)	SD	
I	FAT [%]	24.56 (22.16–26.96)	6.19	23.69 (20.54–26.83)	6.32	28.30 (26.18–30.42)	7.77	**0.023**
	VFAT [level]	10.54 (8.55–12.52)	5.12	8.50 (6.35–10.65)	4.33	10.26 (9.07–11.45)	4.36	0.326
	FFM [%]	75.44 (73.04–77.84)	6.19	76.31 (73.17–79.46)	6.32	71.70 (69.58–73.82)	7.77	**0.023**
	TBW [%]	53.42 (51.55–55.28)	4.81	53.95 (51.57–56.33)	4.78	50.96 (49.36–52.56)	5.86	**0.046**
	PMM [%]	71.66 (69.38–73.94)	5.88	72.47 (69.48–75.45)	6.00	68.09 (66.07–70.11)	7.40	**0.021**
II	FAT [%]	23.78 (21.36–26.19)	6.22	23.56 (20.40–26.72)	6.36	27.52 (25.42–29.61)	7.66	**0.027**
	VFAT [level]	10.39 (8.41–12.37)	5.10	8.50 (6.40–10.60)	4.22	10.06 (8.88–11.24)	4.32	0.390
	FFM [%]	76.25 (73.84–78.66)	6.21	76.49 (73.32–79.65)	6.37	72.47 (70.38–74.57)	7.66	**0.025**
	TBW [%]	53.95 (52.06–55.84)	4.86	54.06 (51.68–56.44)	4.79	51.39 (49.83–52.96)	5.73	0.051
	PMM [%]	72.43 (70.14–74.72)	5.90	72.64 (69.64–75.64)	6.03	68.84 (66.84–70.83)	7.30	**0.025**
III	FAT [%]	23.86 (21.27–26.45)	6.69	23.54 (20.31–26.77)	6.49	27.85 (25.78–29.93)	7.61	**0.017**
	VFAT [level]	10.43 (8.41–12.45)	5.21	8.44 (6.35–10.54)	4.20	10.15 (8.95–11.35)	4.40	0.336
	FFM [%]	76.16 (73.57–78.75)	6.67	76.40 (73.19–79.62)	6.46	72.06 (70.01–74.11)	7.51	**0.017**
	TBW [%]	54.09 (52.11–56.08)	5.12	54.02 (51.62–56.42)	4.82	51.11 (49.57–52–65)	5.63	**0.028**
	PMM [%]	72.35 (69.89–74.81)	6.34	72.56 (69.51–75.61)	6.13	68.41 (66.47–70.36)	7.12	**0.017**

*N*: number of subjects; M: mean; SD: standard deviation; FAT: body fat; VFAT: visceral fat; FFM: fat-free mass; TBW: total body water; PMM muscle mass; *p*: Kruskal–Wallis test; (Kołmogorow–Smirnow test, Shapiro–Wilk test), bold value: statistical significance.

**Table 5:** The changes of body mass components over time.

	Exam I vs. II	Exam II vs. III	Exam I vs. III
Fat	**0.001**	0.205	**0.010**
VFatL	**0.033**	0.399	0.246
FFM	**0.001**	0.077	**<0.001**
TBW	**0.009**	0.280	**0.0270**
PMM	**0.001**	0.058	**<0.001**

FAT: body fat; VFAT: visceral fat; FFM: fat-free mass; TBW: total body water; PMM muscle mass; bold value: statistical significance.

**Table 6:** Multi-factor ANOVA tests for between-subjects effects.

Tested Effect of Factors (ANOVA)	FAT			VFAT			FFM			TBW			PMM		
	Exam I	Exam II	Exam III	Exam I	Exam II	Exam III	Exam I	Exam II	Exam III	Exam I	Exam II	Exam III	Exam I	Exam II	Exam III
Sex	**0.007**	0.062	**0.038**	**0.001**	**0.001**	**0.001**	**0.007**	0.064	**0.038**	**0.007**	0.069	**0.032**	**0.007**	0.061	**0.036**
Age	**0.012**	**0.049**	**0.040**	**<0.001**	**0.001**	**0.001**	**0.012**	**0.048**	0.083	**0.001**	**0.010**	**0.021**	**0.012**	**0.046**	0.083
Type of stroke	0.829	0.968	0.952	0.657	0.631	0.568	0.829	0.984	0.859	0.715	0.919	0.837	0.836	0.979	0.864
Time from stroke	0.057	0.061	**0.029**	**0.017**	**0.022**	**0.010**	0.057	0.060	**0.030**	0.164	0.157	0.076	0.059	0.061	**0.030**
Sex vs.Age	**0.004**	**0.002**	**0.006**	0.104	0.099	0.149	**0.004**	**0.002**	**0.006**	**0.003**	**0.002**	**0.005**	**0.004**	**0.002**	**0.007**
Sex vs.Type of stroke	0.302	0.081	0.0830	0.281	0.219	0.124	0.302	0.076	0.080	0.425	0.130	0.145	0.302	0.074	0.079
Sex vs. Time from stroke	0.055	0.057	0.093	0.654	0.656	0.687	0.055	0.056	0.096	**0.030**	**0.044**	0.085	0.053	0.054	0.100
Age vs. Type of stroke	0.219	0.719	0.526	0.926	0.713	0.795	0.219	0.710	0.751	0.252	0.638	0.698	0.217	0.699	0.748
Age vs. Time from stroke	0.231	0.287	0.201	0.345	0.414	0.358	0.231	0.288	0.202	0.066	0.158	0.111	0.236	0.288	0.212
Type of stroke vs. Time from stroke	0.667	0.255	0.170	0.206	0.155	0.105	0.667	0.256	0.167	0.895	0.474	0.364	0.673	0.256	0.165
Sex vs. Age vs. Type of stroke	0.955	0.424	0.425	0.374	0.281	0.218	0.955	0.443	0.448	0.617	0.713	0.659	0.948	0.446	0.442
Sex vs. Age vs. Time from stroke	0.438	0.636	0.577	0.989	0.953	0.659	0.438	0.634	0.568	0.365	0.591	0.547	0.440	0.631	0.589

FAT: body fat; VFAT: visceral fat; FFM: fat-free mass; TBW: total body water; PMM muscle mass; bold value: statistical significance.

## References

[1] Chen Z., Iona A., Parish S., Chen Y., Guo Y., Bragg F., Yang L., Bian Z., Holmes M.V., Lewington S. (2018). Adiposity and risk of ischaemic and haemorrhagic stroke in 0,5 million Chinese men and women: A prospective cohort study. Lancet Glob. Health.

[2] Vafadar A.K., Côté J.N., Archambault P.S. (2015). Effectiveness of functional electricalstimulation in improving clinical outcomes in the upper arm following stroke: A systematic review and meta-analysis. BioMed Res. Int..

[3] Drużbicki M., Przysada G., Guzik A., Brzozowska-Magoń A., Kołodziej K., Wolan-Nieroda A., Majewska J., Kwolek A. (2018). The efficacy of gait training using a body weight support treadmill and visual biofeedback in patients with subacute stroke: A randomized controlled trial. BioMed Res. Int..

[4] Mao Y.R., Lo W.L., Lin Q., Li L., Xiao X., Raghavan P., Huang D.F. (2015). The effect of body weight support treadmill training on gait recovery, proximal lower limb motor pattern, and balance in patients with subacute stroke. BioMed Res. Int..

[5] Wiszniewska M., Kobayashi A., Milewska D., Szych Z., Członkowska A. (2006). Gender-related differences in risk factors distribution in ischaemic stroke in various age groups. Post Psychiatr. Neurol..

[6] Boehme A.K., Esenwa C., Elkind M.S. (2017). Stroke Risk Factors, Genetics, and Prevention. Circ. Res..

[7] Jabłońska R., Sadowska M., Królikowska A., Haor B., Ślusarz R. (2016). Functional capacity and risk factors and sociodemographic variables of patients after ischemic stroke. Med. Health Sci. Rev..

[8] Willey J.Z., Moon Y.P., Sacco R.L., Greenlee H., Diaz K.M., Wright C.B., Elkind M.S., Cheung Y.K. (2017). Physical inactivity is a strong risk factor for stroke in the oldest old: Findings from a multi-ethnic population (the Northern Manhattan Study). Int. J. Stroke.

[9] Kroll M.E., Green J., Beral V., Sudlow C.L., Brown A., Kirichek O., Price A., Yang T.O., Reeves G.K., Million Women Study Collaborators (2016). Adiposity and ischemic and hemorrhagic stroke. Prospective study in women and meta-analysis. Neurology.

[10] Wang Q., Gao C., Liu H., Li W., Zhao Y., Xu G., Yan C., Lin H., Lang L. (2017). Hypertension modifies the short-term effects of temperature on morbidity of hemorrhagic stroke. Sci. Total Environ..

[11] Deoke A., Deoke S., Saoji A., Hajare S. (2012). Profile of Modifiable and Non-Modifiable Risk Factors in Stroke in a Rural Based Tertiary Care Hospital-A Case Control Study. Glob. J. Health Sci..

[12] Kałużny K., Kałużna A., Kochański B., Cichosz M., Płoszaj O., Pawiłan M. (2016). The influence of neurological rehabilitation on the functioning of patients after ischemic stroke-A retrospective analysis. J. Educ. Health Sport.

[13] Dagenais G.R., Yi Q., Bosch J., Mann J.F., Pogue J., Yusuf S. (2005). Prognostic impact of body weight and abdominal obesity in women and men with cardiovascular disease. Am. Heart J..

[14] Kurth T., Gaziano J.M., Berger K., Kase C.S., Rexrode K.M., Cook N.R., Buring J.E., Manson J.E. (2002). Body mass index and the risk of stroke in men. Arch. Intern. Med..

[15] Kawate K., Kayaba K., Hara M., Kotani K., Ishikawa S.J., Medical School Cohort Study Group (2017). Body mass index and stroke incidence in Japanese community residents: The Jichi Medical School (JMS) Cohort Study. J. Epidemiol..

[16] Zahn K., Linseisen J., Heier M., Peters A., Thorand B., Nairz F., Meisinger C. (2018). Body fat distribution and risk of incident ischemic stroke in men and women aged 50 to 74 years from the general population. The KORA Augsburg cohort study. PLoS ONE.

[17] Twig G., Yaniv G., Levine H., Leiba A., Goldberger N., Derazne E., Ben-Ami Shor D., Tzur D., Afek A., Shamiss A. (2016). Body-Mass Index in 2.3 Million Adolescents and Cardiovascular Death in Adulthood. N. Engl. J. Med..

[18] Csige I., Ujvarosy D., Szabo Z., Lorincz I., Paragh G., Harangi M., Somodi S. (2018). The Impact of Obesity on the Cardiovascular System. J. Diabetes Res..

[19] Pollock A., Baer G., Campbell P., Choo P.L., Forster A., Morris J., Pomeroy V.M., Langhorne P. (2014). Physical rehabilitation approaches for the recovery of function and mobility following stroke. Cochrane Database Syst. Rev..

[20] Yoshimura Y., Wakabayashi H., Bise T., Tanoue M. (2018). Prevalence of sarcopenia and its association with activities of daily living and dysphagia in convalescent rehabilitation ward inpatients. Clin. Nutr..

[21] Chang K.V., Wu W.T., Huang K.C., Han D.S. (2020). Segmental body composition transitions in stroke patients: Trunks are different from extremities and strokes are as important as hemiparesis. Clin. Nutr..

[22] Ryan A.S., Buscemi A., Forrester L., Hafer-Macko C.E., Ivey F.M. (2011). Atrophy and intramuscular fat in specific muscles of the thigh: Associated weakness and hyperinsulinemia in stroke survivors. Neurorehabil. Neural Repair.

[23] English C., Thoirs K., Coates A., Ryan A., Bernhardt J. (2012). Changes in fat mass in stroke survivors: A systematic review. Int. J. Stroke.

[24] Celik B., Ones K., Ince N. (2008). Body composition after stroke. Intern. J. Rehabil. Res..

[25] Coleman E.R., Moudgal R., Lang K., Hyacinth H.I., Awosika O.O., Kissela B.M., Feng W. (2017). Early Rehabilitation After Stroke: A Narrative Review. Curr. Atheroscler. Rep..

[26] Gbiri C.A., Akinpelu A.O. (2013). Relationship between post-stroke functional recovery and quality of life among Nigerian strokesurvivors. Niger. Postgrad. Med. J..

[27] Sheffler L.R., Knutson J.S., Gunzler D., Chae J. (2012). Relationship between body mass index and rehabilitation outcomes in chronic stroke. Am. J. Phys. Med. Rehabil..

[28] Heuschmann P.U., Kircher J., Nowe T., Dittrich R., Reiner Z., Cifkova R., Malojcic B., Mayer O., Bruthans J., Wloch-Kopec D. (2015). Control of main risk factors after ischaemic stroke across Europe: Data from the stroke-specific module of the EUROASPIRE III survey. Eur. J. Prev. Cardiol..

[29] Thompson D., Karpe F., Lafontan M., Frayn K. (2012). Physical activity and exercise in the regulation of human adipose tissue physiology. Physiol. Rev..

[30] Kizer J.R., Biggs M.L., Ix J.H., Mukamal K.J., Zieman S.J., de Boer I.H., Mozaffarian D., Barzilay J.I., Strotmeyer E.S., Luchsinger J.A. (2011). Measures of adiposity and future risk of ischemic stroke and coronary heart disease in older men and women. Am. J. Epidemiol..

[31] Orsatti F.L., Nahas E.A., Nahas-Neto J., Maesta N., Orsatti C.L., Vespoli Hde L., Traiman P. (2010). Association between anthropometric indicators of body fat and metabolic risk markers in post-menopausal women. Gynecol. Endocrinol..

[32] Dubiński A., Zdrojewicz Z. (2006). The Influence of Obesity, Distribution of Fat Tissue and Leptin on Risk of Developing of Cardiovascular Disease in Women. Adv. Clin. Exp. Med..

[33] Fuster J.J., Ouchi N., Gokce N., Walsh K. (2016). Obesity-Induced Changes in Adipose Tissue Microenvironment and Their Impact on Cardiovascular Disease. Circ. Res..

[34] You T., Ryan A.S., Nicklas B.J. (2004). The Metabolic Syndrome in Obese Postmenopausal Women: Relationship to Body Composition, Visceral Fat, and Inflammation. J. Clin. Endocrinol. Metab..

[35] Park H.S., Lee K. (2005). Greater beneficial effects of visceral fat reduction compared with subcutaneous fat reduction on parameters of the metabolic syndrome: A study of weight reduction programmes in subjects with visceral and sub−cutaneous obesity. Diabet. Med..

[36] Zhang H., Tong T.K., Qiu W., Zhang X., Zhou S., Liu Y., He Y. (2017). Comparable effects of high-intensity interval training and prolonged continuous exercise training on abdominal visceral fat reduction in obese young women. J. Diabetes Res..

[37] Staiger H., Tschritter O., Machann J., Thamer C., Fritsche A., Maerker E., Schick F., Häring H.U., Stumvoll M. (2003). Relationship of serum adiponectin and leptin concentrations with body fat distribution in humans. Obes. Res..

[38] Rykała J. (2009). Influence of the body weight on the quality of life and the frequency of complications occurrence of people after a brain stroke. Eur. J. Clin. Exp. Med..

[39] Akazawa N., Harada K., Okawa N., Tamura K., Moriyama H. (2019). Low body mass index negatively affects muscle mass and intramuscular fat of chronic stroke survivors. PLoS ONE.

[40] Kornet M., Głowacka-Mrotek I., Nowacka K., Hagner W. (2017). Upper limb treatment technigues for stroke survivors. J. Educ. Health Sport.

[41] Manners J., Khandker N., Barron A., Aziz Y., Desai S.M., Morrow B., Delfyett W.T., Martin-Gill C., Shutter L., Jovin T.G. (2019). An interdisciplinary approach to inhospital stroke improves stroke detection and treatment time. J. Neurointerv. Surg..

[42] Chan J., Knutsen S.F., Blix G.G., Lee J.W., Fraser G.E. (2002). Water, other fluids, and fatal coronary heart disease: The adventist health study. Am. J. Epidemiol..

[43] Bahouth M.N., Gaddis A., Hillis A.E., Gottesman R.F. (2018). Pilot study of volume contracted state and hospital outcome after stroke. Neurol. Clin. Pract..

[44] Popkin B.M., D’Anci E.K., Rosenberg H.I. (2010). Water hydration and health. Nutr. Rev..

[45] Wiśniewska K., Kurowska E., Okręglicka K. (2014). Effect water intake on body weight. Med. News.

[46] Anton S.D., Martin C.K., Han H., Coulon S., Cefalu W.T., Geiselman P., Williamson D.A. (2010). Effects of stevia, aspartame, and sucrose on food intake, satiety, and postprandial glucose and insulin levels. Appetite.

[47] Vij V.A., Joshi A.S. (2013). Effect of ‘water induced thermogenesis’ on body weight, body mass index and body composition of overweight subjects. J. Clin. Diagn. Res..

[48] Dennis E.A., Dengo A.L., Comber D.L., Flack K.D., Savla J., Davy K.P., Davy B.M. (2010). Water consumption increases weight loss during a hypocaloric diet intervention in middle-aged and older adults. Obesity.

[49] Kalichman L., Rodrigues B., Gurvich D., Israelov Z., Spivak E. (2007). Impact of patient’s weight on stroke rehabilitation results. Am. J. Phys. Med. Rehabil..

